# The diversity of the HLA-E-restricted peptide repertoire explains the immunological impact of the Arg107Gly mismatch

**DOI:** 10.1007/s00251-015-0880-z

**Published:** 2015-11-09

**Authors:** Alexander A. Celik, Thomas Kraemer, Trevor Huyton, Rainer Blasczyk, Christina Bade-Döding

**Affiliations:** Institute for Transfusion Medicine, Hannover Medical School, Medical Park, Feodor-Lynen-Str. 5, 30625 Hannover, Germany

**Keywords:** HLA-E*01:03, Diverse HLA-E peptide repertoire, Non-canonical peptides, Tumor immune escape

## Abstract

Human leukocyte antigen (HLA)-E molecules are potent inhibitors of NK cell-mediated killing. Low in polymorphisms, two alleles are widely expressed among diverse populations: HLA-E*01:01 and HLA-E*01:03. Both alleles are distinguished by one SNP resulting in the substitution Arg107Gly. Both alleles present a limited set of peptides derived from class I leader sequences physiologically; however, HLA-E*01:01 presents non-canonical peptides in the absence of HLA class I molecules. To further assess the functional differences between both alleles, we analyzed the peptide repertoire of HLA-E*01:03 by applying soluble HLA technology followed by mass-spectrometric peptide sequencing. HLA-E*01:03 restricted peptides showed a length of 9–17 amino acids and differed in their biophysical properties, no overlap in the peptide repertoire of both allelic variants could be observed; however, both alleles shared marginal peptides from the same proteomic content. Artificial APCs expressing empty HLA-E*01:01 or E*01:03 molecules were generated and stabilized using cognate HLA class I-derived peptide ligands to analyze the impact of residue 107 within the HLA-E heavy chain on the NKG2/CD94 receptor engagement. Differences in peptide stabilization could be translated to the density and half-life time of peptide-HLA-E molecules on the cell surface that subsequently impacted NK cell inhibition as verified by cytotoxicity assays. Taken together, these data illustrate functional differences of HLA-E allelic variants induced by a single amino acid. Furthermore, the function of HLA-E in pathophysiologic situations when the HLA processing machinery is interrupted seems to be more emphasized than previously described, implying a crucial role for HLA-E in tumor or viral immune episodes.

## Introduction

The human leukocyte antigen (HLA) gene clusters rank among the most polymorphic in humans, and consequently, several thousand alleles coding for functional polypeptides are identified by now (Robinson et al. 2015). Of the three major classes, the HLA class I molecules are expressed on almost every nucleated cell. As they constitute the ligand for CD8^+^ T cells (Bjorkman and Parham 1990), the primary role of HLA class I molecules is to monitor the immunological status of individual cells by scanning the proteomic content. Intracellular processed peptides with a length of 8 to 16 amino acids (AA) (Bade-Doding et al. 2011; Burrows et al. 2006; Rammensee et al. 1993) are loaded onto HLA class I molecules, these trimeric complexes report the health status of the cell. Since most HLA polymorphisms are located in the peptide-binding region (PBR) (Parham et al. 1988), different alleles present potentially different peptides (Bade-Doeding et al. 2007; Bade-Doeding et al. 2004; Badrinath et al. 2012a). Subsequently, every single peptide-HLA (pHLA) complex represents a different ligand for a specific T cell receptor (TCR) on CD8^+^ T cells.

The classical (class Ia) molecules HLA-A, HLA-B, or HLA-C are highly polymorphic, whereas non-classical (class Ib) molecules HLA-E, HLA-F, or HLA-G are more conserved, suggesting a minor role in the presentation of antigen diversity. For HLA-E, only 17 alleles are known to date according to the IMGT/HLA database (Robinson et al. 2015). Such conservation of the polypeptide structure might be correlated to the function of HLA-E to primarily interact with NK cells (Braud et al. 1998; Lee et al. 1998b), immune cells commonly associated with the innate immune system due to their conserved immune receptors.

However, it was shown that certain pHLA-E complexes are ligands for an HLA-E-specific subset of CD8^+^ T cells: HLA-E*01:03^VMAPRTLIL^, peptide source *human cytomegalovirus* (HCMV) UL40 or HLA-Cw3 (Mazzarino et al. 2005; Pietra et al. 2003), or HLA-E*01:01/03^SQAPLPCVL^, peptide source *Epstein–Barr virus* (EBV) BZLF-1 (Romagnani et al. 2002). This indicates that HLA-E acts as an intermediate molecule bridging interactions between adaptive and innate immunity. Of the known HLA-E alleles, only two are widely distributed in different ethnic populations (Felicio et al. 2014). Both alleles differ in only one AA, located outside the PBR at position 107 (Grimsley and Ober 1997; Strong et al. 2003); here, Arg (HLA-E*01:01, HLA-E^R^) is replaced by Gly (HLA-E*01:03, HLA-E^G^). Strong et al. (2003) elucidated that the Arg^107^ > Gly^107^ exchange leads to higher thermal stability when bound to the same peptide, resulting in a more stable expression of HLA-E*01:03 on the cell surface compared to HLA-E*01:01. Higher thermal stability potentially influences the half-life of the molecule as well, prolonging the possible interaction time of the HLA molecule with immune effector cells. However, the functional effects of the AA substitution have not been systematically analyzed.

HLA-E is a ligand for the NKG2/CD94 receptor present on NK cells (Braud et al. 1998; Lee et al. 1998b) as well as on a subset of T cells (McMahon and Raulet 2001; Mingari et al. 1996). Dependent on the NKG2 isoform, the NKG2/CD94 receptor complex conducts either inhibitory (e.g., NKG2A/CD94) or lytic (e.g., NKG2C/CD94) functions (Brooks et al. 1997; Lanier 2008). HLA-E usually presents peptides derived from the leader sequence of other HLA class I molecules (Braud et al. 1997), the presentation of such peptides to the NKG2A/CD94 heterodimer inhibits NK cell-mediated lysis. In this regard, Kaiser et al. (2005) reported that HLA-E bound to the HLA-G leader peptide (VMAPRTLFL) confers a sixfold increased binding affinity to NKG2A/CD94 compared to NKG2C/CD94. Analyzing the structure of HLA-E*01:01^VMAPRTLFL^ bound to NKG2A/CD94, Petrie et al. (2008) elucidated that the specificity of the interaction is dictated by the CD94 subunit, whereas the affinity of the receptor is modulated by the NKG2 subunit. Hereby, CD94 interacts with position p5, p6, and p8 of the HLA-E-bound peptide chain, whereas NKG2A only directly interacts with p5.

However, in recent years, it became evident that HLA-E ligands are not restricted to the leader peptides of HLA class I molecules (Kraemer et al. 2014; Stevens et al. 2001).

More specifically, a peptide derived from gliadin (SQQPYLQLQ) was found to stabilize HLA-E (Terrazzano et al. 2007), whereas for the mouse MHC-E homologue Qa-1 binding of heat shock protein 60 (hsp60) from mouse and bacteria was found by means of random peptide libraries. Consequently, Michaelsson et al. (2002) reported that HLA-E is able to bind a peptide (QMRPVSRVL) derived from the leader sequence of human hsp60, which is upregulated during cellular stress, the HLA-E*01:01^QMRPVSRVL^ as well as the HLA-E*01:03^QMRPVSRVL^ complex failed to inhibit NK cell-mediated lysis via NKG2A/CD94 emphasizing the magnitude of the bound peptide’s sequence in the context of this interaction. By contrast, Wooden et al. (2005) identified a non-leader peptide (ALALVRMLI) derived from the ATP-binding cassette transporter multidrug resistance-associated protein 7 that is capable of inhibiting NK cell-mediated lysis when presented by HLA-E.

The spectrum of peptides that can be selected and presented by HLA-E varies from peptides derived from self-proteomic content to peptides of pathogenic origin. It is known that HCMV, e.g., interferes with HLA class Ia molecule expression or maturation in order to evade recognition by the immune system (Wiertz et al. 1996). To evade detection of infected cells by NK cells due to lack of surface HLA class Ia expression, human cytomegalovirus promotes upregulation of HLA-E and provides a peptide (VMAPRTLIL) derived from HCMV UL40 (Pietra et al. 2003; Tomasec et al. 2000) that mimics the leader sequence of HLA-Cw03 (VMAPRTLIL). Presentation of this peptide by HLA-E prevents NK cell-mediated lysis.

Nattermann et al. (2005a) showed that HLA-E expression is stabilized during HCV infection by the HCV core-derived peptide YLLPRRGPRL and that, additionally, NKG2A is upregulated during chronic HCV infection (Nattermann et al. 2006). Schulte et al. (2009) reported a functional difference for HLA-E^R^ vs. HLA-E^G^ in chronic HCV genotype 2- and 3-positive patients, stating that in such patients, an HLA-E^R/R^ genotype occurs less often. Possibly due to the reduced stability of HLA-E^R^, effective engagement of NKG2A/CD94 is further lowered resulting in a higher susceptibility to NK cell-mediated lysis. On a related note, a treatment-induced clearance of HCV was shown to be associated with the HLA-E^R^ allele (Guzman-Fulgencio et al. 2013).

Lajoie et al. (2006) showed in *human immunodeficiency virus* (HIV)^+^ Zimbabwean women that an HLA-E^G/G^ genotype was associated with a fourfold decreased risk of HIV-1 infection compared to a heterozygous or homozygous HLA-E^R/R^ genotype, possibly due to increased NK cell-mediated lysis of infected cells. In this regard, Nattermann et al. (2005b) were able to identify a peptide (AISPRTLNA) derived from the p24 protein of HIV-1 that resembles the binding motif of other class Ia leader peptides. It is not only capable of binding to HLA-E but also stabilizes its surface expression and facilitates inhibition of NK cell-mediated lysis.

Viral immune escape mechanisms are very diverse, classical HLA class I expression is diminished to avoid the presentation of viral peptides. The opposite holds true for HLA-E, the presentation of viral peptides on HLA-E molecules is desired, and viral-peptide-HLA-E complexes manipulate the expression of NKG2A/CD94 on NK cells, avoiding cell destruction of infected cells due to the NK *missing self*-*recognition*. The balance of preventing viral-peptide-HLA class I presentation on infected cells but facilitating viral-peptide-HLA-E presentation in infected cells is very subtle and requires an accurate immune control system. How that control system operates is not well understood, yet. A first step is the determination of HLA-E-restricted self-peptide repertoires and the interaction of certain pHLA-E complexes with immune effector cells.

HLA-E does not solely interact with the innate immune system but also represents a ligand for the αβTCR of CD8^+^ T lymphocytes (Hoare et al. 2006). Alloreactive CD8^+^ T cells (CTLs) are well-known mediators of graft versus host disease (GvHD) reactions following allogenic transplantation; however, such CTLs can also provide an anti-leukemic effect. This graft versus leukemia (GvL) termed reaction is crucial in eliminating residual malignant cells. However, since NK cells are one of the first lymphocytes to be reconstituted post transplantation, their involvement during GvL reactions is the subject of ongoing research (Cooley et al. 2009; Kroger et al. 2011; Ruggeri et al. 2002; Verneris 2013). Considering that NK cells are the primary immune effectors for HLA-E recognition, such immune interactions directly after transplantation might be of special importance for the overall success of the procedure. The marginal level of polymorphism for HLA-E on one hand and the diversity of interaction with innate or adaptive immune receptors on the other hand lead to the assumption that (i) the peptide repertoire of HLA-E is highly miscellaneous and (ii) the two prevalent occurring allelic subtypes are presumably immunogenic different. During allogenic *hematopoietic stem cell transplantation* (HSCT), for instance, homozygosity for HLA-E*01:03 was shown to benefit patients, possibly due to an improved GvL effect. Homozygosity for HLA-E*01:03 resulted in lower incidence of relapse (Hosseini et al. 2013) compared to patients homozygous for HLA-E*01:01 or heterozygous patients. Additionally, Danzer et al. (2009) showed an increased disease-free survival or a reduction of transplant-related mortality for homozygous carriers of HLA-E*01:03. Moreover, Tamouza et al. (2006) showed that homozygosity for HLA-E*01:03 contributed to the improved survival of patients after genoidentical HSCT. Although the mechanisms are not fully understood, the authors discuss the possible presentation of minor histocompatibility antigens (mHAGs) different from classical mHAGs by HLA-E or the competition in presenting classical mHAGs with classical HLA, however, without inducing T cell activation that would subsequently lower the chance for GvHD.

Recent work identified a novel peptide repertoire in the absence of HLA class I leader peptides for HLA-E*01:01 (Kraemer et al. 2015). HLA molecules themselves and designated components of the peptide loading complex (PLC) represent targets for viral immune evasion. Previous work by Lampen et al. (2013) demonstrated an alternative peptide repertoire for HLA-E in the absence of *functional transporter associated with antigen processing* (TAP), a certain target of viral immune escape since peptide loading of HLA molecules is dependent on the TAP complex. Additionally, HCMV is known to inhibit tapasin (TPN) (Park et al. 2004) as part of the viral immune escape. To further elucidate the differences between both alleles, we determined the peptide repertoire of HLA-E*01:03 in the absence of class I molecules and additionally investigated the peptides presented by both allelic variants in the absence of HLA class I molecules and the absence of TPN in a model cell system. The obtained results provide novel aspects about HLA-E antigen presentation and the maintenance of the single allelic polymorphism which contribute to the understanding of its impact in different clinical situations.

## Material and methods

### Cell lines

HLA^−^/TPN^+^ LCL721.221 (0.221) or HLA^−^/TPN^−^ LCL721.220 (0.220) cell lines were transduced with lentiviral vectors encoding for truncated, soluble forms of HLA-E*01:01 or HLA-E*01:03 (sHLA-E, exon 1–4); HLA^−^/TPN^+^/TAP^−^ T2 cells were transduced with lentiviral vectors encoding for full-length HLA-E*01:01 or HLA-E*01:03 (T2E, exon 1–7); lymphocytes were maintained in RPMI 1640 medium supplemented with 10 % fetal bovine serum (heat inactivated, FBS) and 2 mM l-glutamine. HEK293T cells were maintained in DMEM medium (Life Technologies, Darmstadt, Germany) supplemented with 10 % FBS, 2 mM l-glutamine, 100 U/ml penicillin, 100 μg/ml streptomycin, and 1 mg/ml geneticin (Life Technologies, Darmstadt, Germany). The KIR^−^/NKG2/CD94^+^ NKL (Drexler and Matsuo 2000; Robertson et al. 1996) cell line (kindly provided by C. S. Falk, Hannover Medical School, Germany) was maintained in RPMI 1640 supplemented with 15 % FBS, 2 mM l-glutamine, 1 mM sodium pyruvate, 100 U/ml penicillin, 100 μg/ml streptomycin, and 200 U/ml IL-2. All cell lines were maintained at 37 °C in an atmosphere of 5 % CO_2_.

### Lentiviral transduction of lymphocytes with HLA-E constructs

HLA-E*01:03 inserts were generated via site-directed mutagenesis utilizing the QuikChange® II XL Site-Directed Mutagenesis Kit (Agilent Technologies, Waldbronn, Germany). Using HLA-E*01:01 (Kraemer et al. 2015) as template, the point mutation was induced at c.382A > G with primers HLA-E03-SDM382-S (5′ggg CCC gAC ggg CgC TTC CTC 3′) and HLA-E03-SDM382-AS (5′gAg gAA gCg CCC gTC ggg CCC 3′). The appropriate insert (sHLA-E or mHLA-E) was then ligated into the lentiviral vector pRRL.PPT.SFFV.mcs.pre (Badrinath et al. 2012b), and the construct was verified by sequencing. For virus production, HEK293T cells were transfected utilizing Lipofectamine® 2000 (Life Technologies, Darmstadt, Germany) with the respective construct (10 μg plasmid/1*10^6 cells) in combination with packaging (psPAX2) and envelope (pmD2.G) coding vectors (each 5 μg plasmid/1*10^6 cells). After 24 h, viral particles were harvested, concentrated for 16 h at 10,000 rpm and 4 °C, and consequently used for transduction of lymphocytes. Additionally, freshly transduced cells were treated with 8 μg/ml protamine sulfate (Sigma-Aldrich, St. Louis, USA) and incubated in RPMI 1640 without supplements for 8 h. Following medium exchange, the cells were cultivated in full medium.

### Large-scale production of sHLA-E

For large-scale production and subsequent sequencing of HLA-E bound peptides, the soluble HLA technology as described by Kunze-Schumacher et al. (2014) was deployed. To analyze peptide acquisition by HLA-E in the absence of class I leader peptides, the HLA^−^ LCL 721.221 cell line was transduced with sHLA-E*01:03. To further investigate if peptides are acquired independently of TPN, the HLA^−^/TPN^−^ LCL 721.220 was transduced with sHLA-E*01:03 or E*01:01. Expression of sHLA-E was verified by sandwich-ELISA using mab W6/32 (AbD Serotec®, Puchheim, Germany) as coating antibody and anti-β_2_m/HRP (DAKO, Hamburg, Germany) as detection antibody. Clones with the highest production of sHLA-E molecules were cultivated in bioreactors (CELLine by Integra, Fernwald, Germany) at 37 °C and 5 % CO_2_. Supernatant-containing sHLA-E was collected weekly, centrifuged at 300×*g* for 10 min, and filtered using a 0.45-μm membrane (Millipore GmbH, Schwalbach, Germany). Supernatants were adjusted to pH 8.0 prior purification and purified using NHS-activated HiTrap columns (Life Technologies, Darmstadt, Germany) coupled to mab W6/32 (eBioscience, Germany). Peptide-HLA-E (pHLA-E) complexes were eluted using 100 mM glycine adjusted to pH 2.7 (HCl).

### Sequencing of sHLA-E derived peptides

Affinity-purified pHLA-E complexes were treated with 0.1 % trifluoroacetic acid (TFA) to elute peptides and subsequently filtered using a 10-kD cutoff membrane (Millipore, Schwalbach, Germany). Flow-through containing peptides was subjected to reverse-phase chromatography using an Eksigent nano-LC Ultra 2D HPLC system coupled to an Orbitrap mass spectrometer (Thermo Fisher Scientific, Ulm, Germany). Data of high mass accuracy was analyzed using Mascot software (Hirosawa et al. 1993) as well as the SwissProt and SwissProt human decoy databases.

### HLA-E stabilization assay

HLA-E stabilization assays were carried out using different leader peptides (derived from other HLA class I molecules) known to stabilize both HLA-E alleles (Lee et al. 1998a; Llano et al. 1998; Strong et al. 2003): VMAPRTLFL (HLA-G), VMAPRTLVL (HLA-A*02/23/24/25/26/43/66/68/69), VMAPRTLIL (HLA-C*03), or VMAPRALLL (HLA-C*06:17). These nonameric peptides all differ at their p8. VMAPRALLL possesses a different p6. TAP^−^ T2 cells, transduced with constructs encoding for either HLA-E*01:01 (T2E*01:01) or E*01:03 (T2E*01:03) heavy chain, present empty, unstable HLA-E molecules on the cell surface. These cells were pulsed with the respective peptide by incubating 5 × 10^5^ T2E*01:01 or T2E*01:03 cells with peptides at a concentration of 2 to 500 μM in serum-free RPMI 1640 for 2.5 h at 37 °C and 5 % CO_2_. After incubation for 30 min at 4 °C with the mAb 3D12-APC (eBioscience, Frankfurt, Germany), successful stabilization of pHLA-E complexes was analyzed by flow cytometry (FACS Canto II, BD Biosciences, Heidelberg, Germany).

### Cytotoxicity assay

To determine the influence of distinct pHLA-E complexes on NK cell-mediated cytotoxicity, a flow cytometry-based cytotoxicity assay was performed as previously described (Kraemer et al. 2015). T2E*01:01 or T2E*01:03 cells were labeled with 5 μM CFDA-SE/1 × 10^6^ cells (Life Technologies, Darmstadt, Germany), saturated with selected peptides and used as target cells. Successful peptide loading onto HLA-E molecules was verified using mAb W6/32-APC. At a ratio of 10:1, effector cells (NKL) were incubated with target cells for 4 h. Finally, cells were stained using 7-aminoactinomycin D (7-AAD, BioLegend, San Diego, USA) at a concentration of 2 μg/1 × 10^6^ cells. For analysis, 10,000 CFSE^+^ target cells were recorded and analyzed for 7-AAD positivity (i.e., dead target cells). Cells incubated without effector cells were used to assess spontaneous cell death. Specific lysis mediated by NKL cells was calculated according to the following equation:

$$ \%\mathrm{specific}\;\mathrm{lysis}=100\times \frac{\%\mathrm{dead}\;\mathrm{target}\;\mathrm{get}\;\mathrm{cells}-\%\mathrm{spontaneous}\;\mathrm{dead}\;\mathrm{target}\;\mathrm{cells}}{100-\%\mathrm{spontaneous}\;\mathrm{dead}\;\mathrm{target}\;\mathrm{cells}} $$

## Results

### HLA-E*01:03 presents a diverse set of peptides in the absence of class I leader peptides

**Fig. 1:**
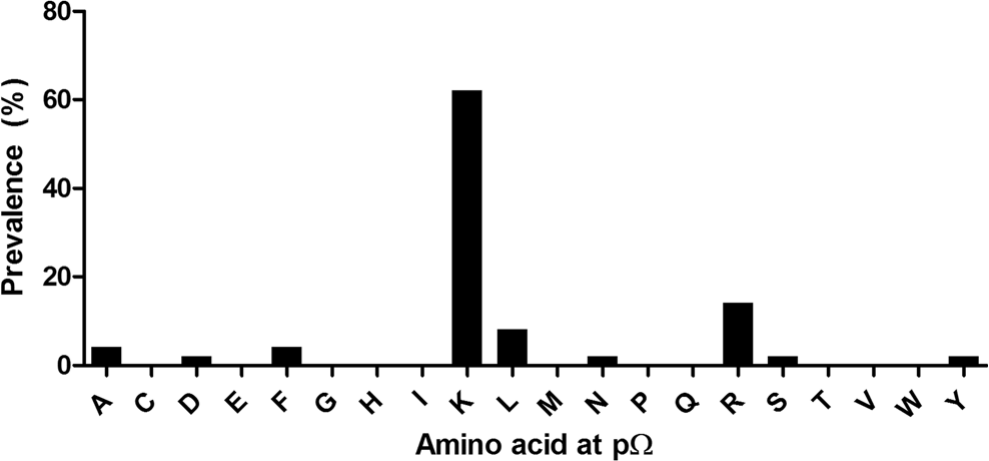
Frequency of AAs at pΩ in peptides derived from sHLA-E*01:03. Peptide-sHLA-E*01:03 complexes were purified from HLA^−^ LCL 721.221 cells. X-axis depicts AA residue at pΩ, y-axis depicts prevalence in the analyzed peptide pool. Peptides were found to be preferably anchored by Lys at pΩ

sHLA-E*01:03 molecules were expressed in LCL 721.221 cells and their bound self-peptides eluted. A set of 56 peptides could be identified (Table 1), ranging from 9 to 17 AAs in length. Of those peptides that showed an extraordinary length of greater than 10 AAs, four 11-mer, seven 12-mer, seven 13-mer, ten 14-mer, nine 15-mer, ten 16-mer, and five 17-mer peptides could be detected. Their cellular origin was diverse, peptides derived from nuclear as well as from cytosolic proteins could be recovered; among these, a set of differentially processed peptides derived from myosin-9, histone H1.5, histone H2A 1-D, and histone H2B 1-J or 1-L was detected (Table 1). Furthermore, peptides of several closely related proteins (60S ribosomal protein L8/L22/L23a/L27/L28/L29/L36/L38, l-acetate dehydrogenase A or B chain) could be identified. For p2, no specific anchor could be identified; however, peptides were preferentially anchored by the positively charged Lys (62 %) or to a lesser extent by the positively charged Arg (14 %, Fig. 1) at pΩ.

**Table 1:** Ligands of HLA-E*01:03 derived from HLA^−^/TPN^+^ LCL 721.221

HLA-E*01:03		
Sequence	Length	Origin
HAVSEGTKAVTKYTSSK	17	Histone H2B type 1-L
PAETATPAPVEKSPAKK	17	Histone H1.5
AYVRLAPDYDALDVANK	17	60S ribosomal protein L23a
HAVSEGTKAVTKYTSAK	17	Histone H2B type 1-J
AVSDGVIKVFNDMKVRK	17	Cofilin-1
HAVSEGTKAVTKYTSS	16	Histone H2B type 1-L
QLLQANPILEAFGNAK	16	Myosin-9
KSADTLWDIQKDLKDL	16	l-lactate dehydrogenase B chain
AYVRLAPDYDALDVAN	16	60S ribosomal protein L23a
HAVSEGTKAVTKYTSA	16	Histone H2B type 1-J
TGLIKGSGTAEVELKK	16	Pyruvate kinase isozymes M1/M2
VSDGVIKVFNDMKVRK	16	Cofilin-1
ASGNYATVISHNPETK	16	60S ribosomal protein L8
TAEILELAGNAARDNK	16	Histone H2A type 1-D
HAVSEGTKAVTKYTSA	16	Histone H2B type 1-J
PAPVEKSPAKKKATK	15	Histone H1.5
SADTLWDIQKDLKDL	15	l-lactate dehydrogenase B chain
TGLIKGSGTAEVELK	15	Pyruvate kinase isozymes M1/M2
KSADTLWGIQKELQF	15	l-lactate dehydrogenase A chain
HGSYEDAVHSGALND	15	T-complex protein 1 subunit alpha
SDGVIKVFNDMKVRK	15	Cofilin-1
AGNLGGGVVTIERSK	15	60S ribosomal protein L22
AQAAAPASVPAQAPK	15	60S ribosomal protein L29
PRKIEEIKDFLLTAR	15	60S ribosomal protein L38
SEGTKAVTKYTSSK	14	Histone H2B type 1-L
VLKQVHPDTGISSK	14	Histone H2B type 1-L
SWTAADTAAQITQR	14	HLA class I histocompatibility antigen, Cw-1 alpha chain
FISVGYVDDTQFVR	14	HLA class I histocompatibility antigen, Cw-1 alpha chain
NIDDGTSDRPYSHA	14	60S ribosomal protein L27
VLKQVHPDTGISSK	14	Histone H2B type 1-J
RKTVTAMDVVYALK	14	Histone H4
SADTLWGIQKELQF	14	l-lactate dehydrogenase A chain
ASAETVDPASLWEY	14	Fascin
TVVNKDVFRDPAL	13	60S ribosomal protein L27
KTVTAMDVVYALK	13	Histone H4
EGIPALDNFLDKL	13	Elongation factor 2
RVTIMPKDIQLAR	13	Histone H3.3C
PVAVMAESAFSFK	13	COP9 signalosome complex subunit 8
QTVAVGVIKAVDK	13	Elongation factor 1-alpha 1
ILELAGNAARDNK	13	Histone H2A type 1-D
GTGASGSFKLNK	12	Histone H1.5
KQVHPDTGISSK	12	Histone H2B type 1-J
VGGTSDVEVNEK	12	60 kDa heat shock protein, mitochondrial
NSVVEASEAAYK	12	14-3-3 protein eta
ALRYPMAVGLNK	12	60S ribosomal protein L36
SLVSKGTLVQTK	12	Histone H1.5
PELAKSAPAPK	11	Histone H2B type 1-L
SEMEVQDAELK	11	Proliferation-associated protein 2G4
QTYSTEPNNLK	11	60S ribosomal protein L28
PMFIVNTNVPR	11	Macrophage migration inhibitory factor
AGFAGDDAPR	10	Actin, cytoplasmic 1
RVNAGTLAVL	10	von Willebrand factor A domain-containing protein 8
IGQSKVFFR	9	Myosin-9

sHLA-E*01:03 molecules were purified from LCL 721.221 and bound peptides sequenced. Given is the sequence and origin of the peptide fragment. Peptide length ranged from 9 to 17 AAs

### HLA-E*01:03 restricted peptides differ substantially from those presented by HLA-E*01:01

Comparing the peptide repertoire of HLA-E*01:03 acquired in the absence of class I molecules to that of HLA-E*01:01 (Kraemer et al. 2015), no overlap in the peptide repertoire could be detected. Both alleles do not share the same peptidome; however, they present peptides from the pool of closely related protein subtypes, histones H2A and H2B (Table 2). Interestingly, one Hsp60-derived 12-mer peptide (VGGTSDVEVNEK) restricted by HLA-E*01:03 could be identified, the signal peptide of Hsp60 bears similarities to class I leader peptides and was previously described to stabilize HLA-E (Michaelsson et al. 2002).

**Table 2:** HLA-E*01:01 and E*01:03 present different peptides of closely related histone subtypes

Sequence	Length	Origin	Allele	Peptide-source	Reference
PKKTESHHKAKGK	13	Histone H2A type 3	E*01:01	0.221	Kraemer et al. (2015)
AAVLEYL	7	Histone H2A type 2-B	E*01:01	0.221	(Kraemer et al. (2015)
TAEILELAGNAARDNK	16	Histone H2A type 1-D	E*01:03	0.221	
ILELAGNAARDNK	13	Histone H2A type 1-D	E*01:03	0.221	

Depicted are peptides derived from histone H2A, eluted from HLA-E*01:01 or E*01:03 expressed in LCL 721.221 (0.221)

### HLA-E*01:01 and HLA-E*01:03 acquire peptides in the absence of TPN

A certain set of peptides could be acquired from HLA^−^/TPN^−^ LCL 721.220 cells (Table 3). Nineteen peptides ranging from 8 to 25 AAs in length and ten peptides ranging from 9 to 17 AAs in length were eluted from sHLA-E*01:01 or sHLA-E*01:03, respectively. Both HLA-E variants select and load peptides via a non-classical TPN-independent peptide loading pathway; however, no shared peptides could be identified. Furthermore, no specific anchor at pΩ could be determined.

**Table 3:** Ligands of HLA-E*01:01 and E*01:03 derived from HLA^−^/TPN^−^ LCL 721.220

Sequence	Length	Origin
HLA-E*01:01		
AQAAAPASVPAQAPKRTQAPTKASE	25	60S ribosomal protein L29
KLEKEEEEGISQESSEEEQ	19	High mobility group protein HMG-I/HMG-Y
GDRSEDFGVNEDLADSDAR	19	Annexin A1
VAPEEHPVLLTEAPLNPK	18	Actin, cytoplasmic 1
STAGDTHLGGEDFDNR	16	Heat shock cognate 71 kDa protein
KVPQVSTPTLVEVSR	15	Serum albumin
PDPAKSAPAPKKGSK	15	Histone H2B type 1-H
LQAEIEGLKGQR	12	Keratin, type II cytoskeletal 8
PDPAKSAPAPK	11	Histone H2B type 1-H
PELAKSAPAPK	11	Histone H2B type 1-L
PEPVKSAPVPK	11	Histone H2B type 1-M
AAPATRAAL	9	Solute carrier family 15 member 4
SAPSRATAL	9	BTB/POZ domain-containing protein KCTD18
ILNFPPPP	8	Caprin-2
IAPTGHSL	8	Septin-6
ISPHGNAL	8	ATP-dependent Clp protease ATP-binding subunit clpX-like, mitochondrial
HLA-E*01:03		
ALAGCHLEDTQRKLQKG	17	Polyamine-modulated factor 1-binding protein 1
MQLITRGKGAGTPNLI	16	Isthmin-1
KMKLRNTVHLSYLTV	15	Taste receptor type 2 member 50
CRASQTISSYLDWYQ	15	Ig kappa chain V-I region OU
PAALTNKGNTVFA	13	Intraflagellar transport protein 88 homologue
WTPGPSAGVTGIA	13	Mucin-19
ILRTIGKEAF	10	Trafficking protein particle complex subunit 8
RSCGYACTA	9	Isthmin-1
FPNGFSFIH	9	Sushi, von Willebrand factor type A, EGF, and pentraxin domain-containing protein 1
SHGPYIKLI	9	Major facilitator superfamily domain-containing protein 2A

sHLA-E*01:03 molecules were purified from LCL 721.220 and bound peptides sequenced. Given is the sequence and origin of the peptide fragment. Peptide length ranged from 8 to 25 AAs

### HLA-E*01:01 and HLA-E*01:03 present peptides of closely related protein isoforms

When comparing the peptides presented by either allele, derived from LCL 721.220, no peptide overlap was detectable. However, comparing the peptides derived from LCL 721.220 cells presented by HLA-E*01:01 and the peptides derived from LCL 721.221 cells presented by HLA-E*01:03, a shared protein origin becomes apparent. Nevertheless, the presented peptide fragments differ between the two HLA-E variants, the notable exception being PELAKSAPAPK, derived from histone H2B 1-L, that is present by HLA-E*01:01 as well as E*01:03 (Table 4).

**Table 4:** Comparison of HLA-E*01:01 and E*01:03 restricted peptides derived from either LCL 721.220 (0.220) or LCL 721.221 (0.221) cells

Sequence	Length	Origin	Allele	Peptide-source
AQAAAPASVPAQAPKRTQAPTKASE	25	60S ribosomal protein L29	E*01:01	0.220
AQAAAPASVPAQAPK	15	60S ribosomal protein L29	E*01:03	0.221
AYVRLAPDYDALDVANK	17	60S ribosomal protein L23a	E*01:03	0.221
AYVRLAPDYDALDVAN	16	60S ribosomal protein L23a	E*01:03	0.221
ASGNYATVISHNPETK	16	60S ribosomal protein L8	E*01:03	0.221
AGNLGGGVVTIERSK	15	60S ribosomal protein L22	E*01:03	0.221
PRKIEEIKDFLLTAR	15	60S ribosomal protein L38	E*01:03	0.221
NIDDGTSDRPYSHA	14	60S ribosomal protein L27	E*01:03	0.221
TVVNKDVFRDPAL	13	60S ribosomal protein L27	E*01:03	0.221
ALRYPMAVGLNK	12	60S ribosomal protein L36	E*01:03	0.221
QTYSTEPNNLK	11	60S ribosomal protein L28	E*01:03	0.221
PDPAKSAPAPKKGSK	15	Histone H2B type 1-H	E*01:01	0.220
PDPAKSAPAPK	11	Histone H2B type 1-H	E*01:01	0.220
PELAKSAPAPK	11	Histone H2B type 1-L	E*01:01	0.220
PEPVKSAPVPK	11	Histone H2B type 1-M	E*01:01	0.220
PELAKSAPAPK	11	Histone H2B type 1-L	E*01:03	0.221
KQVHPDTGISSK	12	Histone H2B type 1-J	E*01:03	0.221
VLKQVHPDTGISSK	14	Histone H2B type 1-J	E*01:03	0.221
SEGTKAVTKYTSSK	14	Histone H2B type 1-L	E*01:03	0.221
HAVSEGTKAVTKYTSA	16	Histone H2B type 1-J	E*01:03	0.221
HAVSEGTKAVTKYTSS	16	Histone H2B type 1-L	E*01:03	0.221
HAVSEGTKAVTKYTSAK	17	Histone H2B type 1-J	E*01:03	0.221
HAVSEGTKAVTKYTSSK	17	Histone H2B type 1-L	E*01:03	0.221
HAVSEGTKAVTKYTSA	16	Histone H2B type 1-J	E*01:03	0.221
VAPEEHPVLLTEAPLNPK	18	Actin, cytoplasmic 1	E*01:01	0.220
AGFAGDDAPR	10	Actin, cytoplasmic 1	E*01:03	0.221

Both alleles present similar peptides from certain protein isoforms

### HLA-E*01:01 and HLA-E*01:03 impact differently on NK cell inhibition

**Fig. 2:**
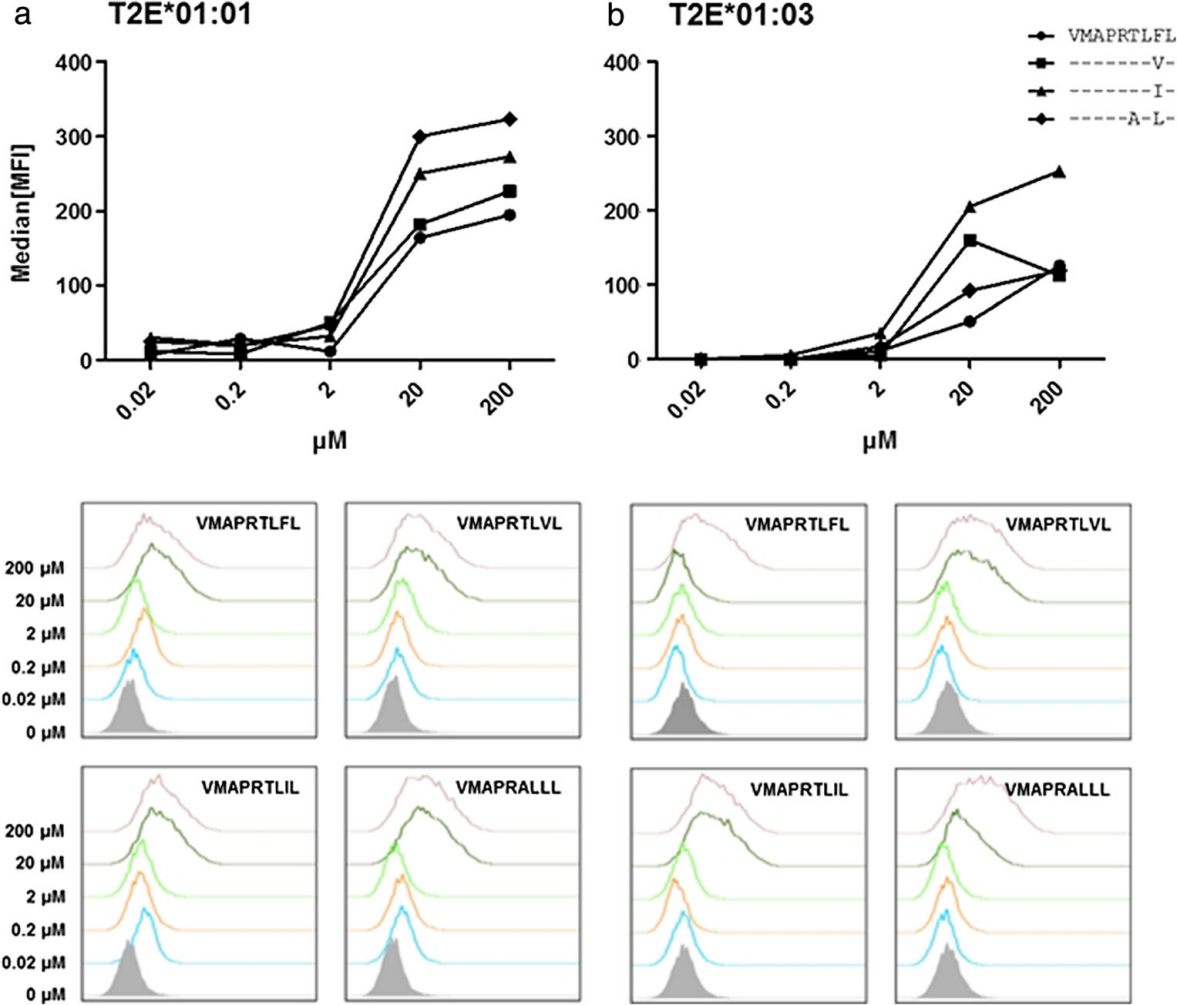
pHLA-E stabilization on T2E cells. Flow cytometric analysis of T2E*01:01 or T2E*01:03 stained with anti-HLA-E-APC conjugated mAb. Depicted is the average of three experiments. **a** T2E saturation was achieved with ≥20 μM of peptide. HLA-E*01:01^VMAPRALLL^ showed the highest stability. **b** T2E saturation was achieved with ≥20 μM of peptide, for HLA-E*01:03^VMAPRTLIL^ showed the highest stability. Saturation was reached with 200 μM of peptide for both alleles

HLA-E molecules are protective immune proteins when bound to HLA class I leader peptides. However, HLA-E*01:01 and HLA-E*01:03 showed a highly diverse peptide repertoire in the absence of class I molecules, suggesting a functional difference when HLA class I is absent. To systematically assess the immunological differences of these alleles, HLA-E*01:01 or E*01:03, each bound to the same peptide were analyzed; for this assay, previously described HLA-E allele-independent restricted HLA class I leader peptides were applied. For stabilization of pHLA-E complexes, the peptides were titrated (Fig. 2). Both alleles bound the HLA-G-derived leader peptide VMAPRTLFL the least efficient. HLA-E*01:01 bound VMAPRALLL (HLA-C*06) most efficiently while E*01:03 bound VMAPRTLIL (HLA-C*03) most efficiently. Both alleles were saturated using similar peptide concentrations.

**Fig. 3:**
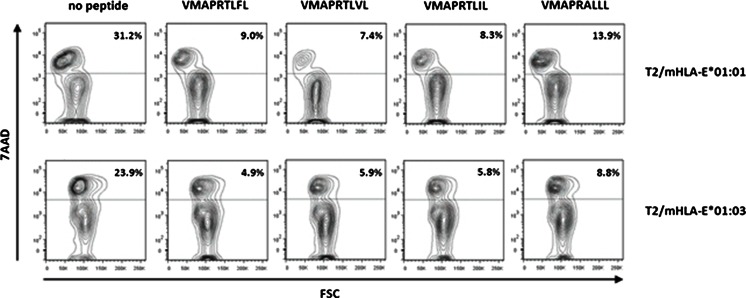
HLA-E-leader peptide complexes confer protection against NK cell mediated lysis. Cytotoxicity assay showing the percentage of lysed target cells. Depicted is the average of three independent experiments, shown FACS plots are exemplary. T2E*01:01 and T2E*01:03 cells were lysed in the absence of peptide. *Top panel*: T2E*01:01^VMAPRTLVL^ showed decreased cytotoxicity (7.4 %) compared to T2E*01:01^VMAPRTLFL^ (9.0 %), T2E*01:01^VMAPRTLIL^ (8.3 %), or T2E*01:01^VMAPRALLL^ (13.9 %). *Bottom panel*: T2E*01:03^VMAPRTLFL^ showed decreased cytotoxicity (7.4 %) compared to T2E*01:03^VMAPRTLVL^ (9.0 %), T2E*01:03^VMAPRTLIL^ (8.3 %), or T2E*01:03^VMAPRALLL^ (13.9 %). Overall cytotoxicity was decreased in T2E*01:03 in comparison to T2E*01:01

To further assess the impact of the 107 mismatch on the interaction with NKG2A/CD94, peptide saturated T2E cells were used as target cells in a cytotoxicity assay. As effector cells, the KIR^−^ and NKG2A^+^, NKG2C^+^, and CD94^+^ NK cell line NKL were used and the amount of lysed target cells analyzed (Fig. 3). T2E cells not bound to a peptide were lysed by NK cells whereas stabilization with different leader peptides led to decreased cytotoxicity. Moreover, each leader peptide-HLA-E complex had different impacts on the level of mediated protection. VMAPRALLL showed higher cytotoxicity than VMAPRTLVL (HLA-A*02), VMAPRTLFL, or VMAPRTLIL. The highest protection was conferred by VMAPRTLVL on T2E*01:01; however, the highest protection on T2E*01:03 was provided by HLA-E*01:03^VMAPRTLFL^, even by its lower density of surface pHLA-E complexes (Fig. 2). Cytotoxicity was found to be decreased for T2E*01:03 cells in comparison to T2E*01:01 cells; however, overall both alleles conferred protection against NK cell-mediated lysis.

## Discussion

HLA-E is a low-polymorphic, non-classical HLA class I molecule. HLA-E*01:01 and E*01:03 frequency is balanced between different populations (Grimsley and Ober 1997). The 107Arg > Gly mismatch separating HLA-E*01:01 and E*01:03 is located in a loop region of the α2-domain outside the peptide binding region and is therefore thought not to influence the conformation of the peptide-binding groove (Strong et al. 2003). Nevertheless, donor homozygosity of E*01:03 was shown to be associated with improved HSCT outcome (Danzer et al. 2009), while donor homozygosity of E*01:01 was not (Tamouza et al. 2006). Both alleles bind nonameric peptides derived from the leader sequence of classical HLA class I molecules under healthy physiological conditions (Lee et al. 1998a). However, HLA-E also binds peptides of diverse origins as shown by Stevens et al. (2001) through random peptide libraries and Lampen et al. (2013) who demonstrated that HLA-E presents diverse peptides even in the absence of functional TAP, this situation occurs for instance during viral infections (Ressing et al. 2005; Vambutas et al. 2001) or in cancerous tissues (Seliger et al. 1998; Seliger et al. 2000). These peptides do not maintain the binding motif of peptides derived from the classical HLA class I leader sequences.

In the present study, we aimed to investigate functional differences between both HLA-E alleles by assessing the repertoire of HLA-E*01:03-acquired peptides in the absence of HLA class I molecules and compared their features with previously identified HLA-E*01:01 peptides from the same proteomic content. Kraemer et al. (2015) showed that HLA-E*01:01 presents peptides of non-canonical length in the absence of HLA class I molecules, displaying variation in length and sequence, and no specific anchoring motifs could be determined.

As given in Table 1, E*01:03 presents non-canonical peptides of various lengths. Analysis of the peptide sequences did not reveal an anchor motif at p2; however, peptides were preferably anchored by Lys at pΩ. Notably, among the identified peptides, few can be assigned to the heavy chain of HLA-Cw1. This might be due to the fact that the HLA-C locus is diminished but not completely abrogated in LCL 721.221 cells leaving the possibility of remaining transcripts as specified by Steinle and Schendel (1994).

Comparing the HLA-E*01:03-restricted peptides to those of E*01:01 (Kraemer et al. 2015), it becomes obvious that the peptide repertoire of both alleles greatly differs in the absence of class I molecules. Both alleles present peptides of different cellular origin, however, both variants share peptide fragments of one particular protein family, histone H2A subtypes; here, the presented peptide fragments differ in length and sequence. This observation suggests that both allelic variants differ in their function and the single mismatch at position 107 causes an alteration of the peptide repertoire. Presumably, this mismatch induces a subtle structural transformation of the alpha 2 helix or a structural-based altered affinity of the peptide receptive HLA-E/β_2_m complex during the peptide loading process could be responsible for the divergent set of presented peptides.

To further specify the functional impact of both HLA-E variants, we utilized a model cell system that mirrors a functional defect in the PLC in addition to abolished HLA class I expression. Therefore, we identified the peptide repertoires of both HLA-E variants in the TPN^−^ and HLA^−^ LCL 721.220 cell line.

TPN is a dedicated part of the PLC and as such represents a potent target for viruses to escape immune recognition; e.g., HCMV inhibits TPN through its immune evasion protein US3 (Park et al. 2004). In addition to the previous findings of Kraemer et al. (2015), we could demonstrate that HLA-E*01:01 as well as E*01:03 acquires peptides derived from intracellular proteins despite the lack of HLA class I molecules and TPN, implying a role for HLA-E in viral immunity. In comparison to the analyzed peptide spectrum of HLA-E variants in B-lymphoblastic cells, we could confirm the functional disparity between both alleles. Comparing the total peptide repertoire of both HLA-E alleles presented in HLA^−^/TPN^−^ cells, it can be observed that overlapping peptide fragments only occur in peptides derived from HLA^−^/TPN^−^ (E*01:01) and HLA^−^ (E*01:03) cells. This highlights that both alleles bind distinct peptides in the presence of TPN; however, its absence impacts HLA-E*01:01 peptide selection as it is shown, e.g., for the peptide PELAKSAPAPK derived from the histone H2B type 1-L protein that is presented by both alleles yet derived from different proteomic content (Table 4). Looking further at the location within the original protein, the presented peptides are not derived from either the N- or C-terminus in the majority of the cases. These findings are similar to the results by Oliveira et al. (2010), who analyzed the peptide repertoire of the HLA-E mouse homologue Qa-1^b^ in the presence and absence of functional TAP. Interestingly, among the peptides presented in TAP^−^ cells, there are also peptides presented from histone H2B. Here, even the AA sequence is equal to that of the peptides bound to E*01:03 from LCL 721.221. However, apart from these peptide sequences, there are also peptides presented from cofilin-1 or the elongation factor 1, albeit the presented fragment is different. These similarities in selection and presentation of unusual peptides by HLA-E or Qa-1^b^ in a pathological situation, in particular with major defects in the PLC machinery, encourage the view of a specialized role for HLA-E in certain diseases.

To compare both alleles for their immunological impact in a physiological situation, the potential to inhibit NK cell cytotoxicity by pHLA-E complexes was assessed. Peptides known to be cognate ligands for both alleles were used. Stable pHLA-E complexes were achieved using different leader peptides derived from HLA class I molecules; however, the different peptides were differentially able to stabilize HLA-E. Eventually, the overall protective potential was shown to be greater for HLA-E*01:03 complexes since more target cells presenting HLA-E*01:01 complexes than target cells presenting E*01:03 complexes have been lysed when both alleles were bound to the same peptide (Fig. 3). Here, the sole functional impact of residue 107 became obvious, when both alleles interact differentially with the cognate NKG2/CD94 NK cell heterodimer. Potentially, a small shift in the overall structure of the PBR may be responsible for such different interaction, even though the mismatch is located in a loop region. In the case of HLA-B*44:02 and B*44:03, it was shown that the Asp156Leu exchange results in a non-permissive mismatch (Badrinath et al. 2012b; Macdonald et al. 2003) through a conformational change of the binding cleft. It is conceivable that the Arg107Gly exchange similarly impacts the overall structure of HLA-E, leading to a slightly different conformation at, for instance, His155, where the α2-domain directly interacts with the NKG2A subunit (Petrie et al. 2008). This could result in an altered affinity to the NKG2A/CD94 heterodimer and thus interfere with NK cell recognition. Nevertheless, further structural studies are necessary to elucidate on that, preferably using high-resolution structures of both alleles bound to the same peptide.

Taken together, these data suggest that even though both alleles are separated only by one mismatch in a loop region, this subtle difference impacts the structure in a way that changes the overall behavior of the molecule. As seen in Fig. 1, HLA-E*01:03 exhibits a strong preference for Lys at the pΩ position and as such may be less variable in the F-pocket than E*01:01. The unusual selection of Lys as an anchor is similar to HLA-B*44:35 that presents longer peptides preferentially anchored by Lys at the C-terminus (Badrinath et al. 2014). This restriction of the pΩ anchor (Fig. 1), at least for the peptide repertoire of HLA-E*01:03, highlights the predicted loop-like peptide conformations formed by the flexible middle part of such long peptides when bound in the PBR of HLA-E*01:01 as described by Kraemer et al. (2015). The outer loop mismatch at position 107 indirectly influences the C-terminal AA selection of the F-pocket. The observation that an outer pocket or outer loop position orchestrates distinct pocket specificity has been observed previously (Bade-Doding et al. 2011; Elamin et al. 2010; Huyton et al. 2012). Moreover, the impact of the single polymorphism on HLA-E function under healthy conditions is more or less little as seen by the differences in NK cell inhibition by HLA class I-derived peptides bound to either HLA-E allele. To maintain this heterozygous HLA-E haplotype among diverse populations, a greater functional impact is likely. Our results provide new insights in the role of HLA-E-driven immune responses displayed by the broadened variations of allele-specific peptide ligands. These results may also help to understand the diverging outcome after HSCT for HLA-E*01:03 HSC homozygous donor. However, further identification of HLA-E peptide repertoires in pathological situations of primary cells is needed for the development of novel therapeutic concepts.
